# Contemporary sequential segmental approach to congenital heart disease using four-dimensional magnetic resonance imaging with ferumoxytol: an illustrated editorial

**DOI:** 10.3389/fcvm.2023.1107399

**Published:** 2023-07-04

**Authors:** Shi-Joon Yoo, Gregory Perens, Kim-Lien Nguyen, Takegawa Yoshida, Ankavipar Saprungruang, Glen S. Van Arsdell, J. Paul Finn

**Affiliations:** ^1^ Department of Diagnostic Imaging, Hospital for Sick Children, Toronto, ON, Canada; ^2^ Division of Cardiology, Department of Paediatrics, Hospital for Sick Children, Toronto, ON, Canada; ^3^ Department of Pediatric Cardiology, Mattel Children's Hospital, David Geffen School of Medicine at UCLA, Los Angeles, CA, United States; ^4^ Diagnostic Cardiovascular Imaging Section, Department of Radiological Sciences, David Geffen School of Medicine at UCLA, Los Angeles, CA, United States; ^5^ Division of Cardiology, David Geffen School of Medicine at UCLA and VA Greater Los Angeles Healthcare System, Los Angeles, CA, United States; ^6^ Department of Pediatric Cardiology, Cardiac Center, King Chulalongkorn Memorial Hospital, Bangkok, Thailand; ^7^ Department of Cardiothoracic Surgery, David Geffen School of Medicine at UCLA, Los Angeles, CA, United States

**Keywords:** MR angiography, 4D imaging, 4D MUSIC, ferumoxytol, segmental approach, congenital heart disease

## Abstract

The ferumoxytol-enhanced 4D MR angiography with MUSIC (Multiphase Steady State Imaging with Contrast) technique provides a single data set that captures dynamic cardiovascular anatomy and ventricular function at the same time. Homogeneous opacification of all cardiovascular structures within the imaging volume allows full sequential segmental approach to the congenital heart diseases without any blind spots. The complex systemic and pulmonary venous anatomy is particularly well captured in the MUSIC. Cinematographic display of multiplanar sectional and 3D volume images is helpful in the morphological identification of the cardiac chambers, the assessment of the dynamic nature of the ventricular outflow tracts, and the assessment of the coronary arterial origins and courses.

## 1. Introduction

The sequential segmental approach is a well-established, systematic approach to the diagnosis of congenital heart diseases (CHD) (1, 2). It is particularly important in those cases having abnormal situs or abnormal segmental connections. While two-dimensional (2D) and three-dimensional (3D) imaging is traditionally used for sequential segmental approach, multiphase 3D imaging also known as four-dimensional (4D) imaging has increasingly been used for cardiovascular assessment. While 4D imaging is possible in ultrasound imaging, echocardiographic 4D imaging is limited by its inherent small field of view and abundant artifact from air and bones. 4D imaging with computed tomography (CT) requires significantly increased amount of radiation as compared to 3D imaging and its temporal resolution is limited by the gantry rotation time of the given scanner (3). In addition, CT generally produces non-uniform contrast in different chambers and vascular structures due to contrast bolus dynamics.

The contrast-enhanced magnetic resonance (MR) technique known as 4D MUSIC (Multiphase Steady State Imaging with Contrast) acquires data continuously over several minutes during uninterrupted positive pressure ventilation, offering near-perfect respiratory gating (4–8). When used with the contrast agent ferumoxytol (Feraheme, Covis Pharmaceuticals, Waltham, MA, USA), MUSIC produces high-definition 3D images that span the entire cardiac cycle, with immediate inline reconstruction. Ferumoxytol is an ultrasmall superparamagnetic iron oxide nanoparticle that highlights the blood pool with MR due to its high T1 relaxivity. It is proven safe with a low incidence of significant side effects when it is infused slowly with close monitoring of the vital signs (9–11). Ferumoxytol is particularly helpful in patients with a high risk for nephrogenic systemic fibrosis (NSF) linked to the use of gadolinium-based contrast agents (9–11). In patients with renal failure, ferumoxytol can not only be safe but also therapeutic for iron deficiency. To date, limited access to ferumoxytol for off-label diagnostic use, as well as its high cost, have posed obstacles to the more widespread utilization of the 4D MUSIC technique. However, the hope is that these limitations will subside and at the time of writing, a generic version of ferumoxytol (Sandoz, Basel, Switzerland) has become available in the U.S., already reducing cost for both generic and brand formulations. A Chinese formulation of ferumoxytol has recently been developed and is undergoing clinical evaluation for diagnostic use within mainland China (12). It seems highly likely that an agent with the proven advantages of ferumoxytol will ultimately earn global access.

Owing to its high T1 relaxivity and long intra-vascular half-life (approximately 15 h), ferumoxytol supports homogeneous opacification of all cardiac chambers and vascular structures for a prolonged period of 3D MR data acquisition that is typically performed with electrocardiographic (ECG) gating and respiration navigation. MUSIC images are acquired with isotropic spatial resolution for accurate 2D and 3D reformation. Maximum intensity projection (MIP) and volume rendering (VR) can be used to view images in an infinite number of imaging planes or views for both anatomical and functional assessment, without restrictions on orientation or view angle. While 4D MUSIC with ferumoxytol allows high quality dynamic 3D visualization of the beating heart, it also allows 2D cine planar reformation that is analogous to prescription of conventional 2D cine imaging planes. This property shifts the time and labor from the acquisition phase in a scan room to the post-processing phase in a reading room. Therefore, it results in shortened examination times relative to traditional approaches but with higher resolution and without repeated breath holding. Furthermore, the acquisition protocol is independent of anatomic and structural complexity, and the same protocol applies to all patients. Therefore, the total examination time for 4D MUSIC MR is potentially much shorter than the time required for conventional MR approaches that rely on sequential 2D breath-held cine and single-phase contrast-enhanced MR angiography.

Rotating Cartesian k-space (ROCK) MUSIC is a further development of the MUSIC technique that does not require external signals for respiratory and cardiac gating (13). Another promising technique is the free-running, five-dimensional (5D) MR approach described by Roy et al. (14). The free-running technique reconstructs 3D image sets at both multiple cardiac phases and multiple respiratory phases. At the time of writing, a current drawback of ROCK MUSIC and 5D free-running techniques is the requirement for offline and interactive image reconstruction, limiting practical widespread use. It should be noted that, although 4D and 5D techniques offer advantages over more conventional methods, highly diagnostic studies can be performed using, for example, a combination of 3D steady state free precession (SSFP) cine and dual-phase contrast enhanced MR angiography (15) or a non-contrast 3D technique such as MTC-BOOST (16). MTC-BOOST generates both a 3D bright blood set and a complementary 3D dark blood set by reconstructing magnitude-only and phase-sensitive images respectively, following an inversion magnetization preparation.

4D MUSIC MR is well suited for sequential segmental approach to complex congenital heart diseases because of its wide field of view without any blind spots, homogeneous opacification of all cardiovascular structures, and dynamic demonstration of the cardiovascular anatomy as well as function. This imaging essay illustrates the utility and major advantages of 4D MUSIC MR for the sequential segmental approach to CHD using select case examples with Supplemental video clips. The steps of segmental approach are summarized in Table 1.

**Table 1:** Basic steps in sequential segmental approach to congenital heart diseases.

Step 1	Determination of the abdominal, bronchopulmonary and atrial situs, and the heart position
Step 2	Identification of the cardiac chambers and arterial trunks by the morphological criteria
Step 3	Assessment of the relationship between the components of each segment
3–1 3–2 3–3	Atrial relationship (atrial situs): Already determined at Step1 Ventricular relationship (ventricular loop pattern) Arterial relationship (position of the aortic root relative to the pulmonary arterial trunk)
Step 4	Assessment of the intersegmental connections
4–1 4–2 4–3	Systemic and pulmonary venous connections to the atria Atrioventricular connections Ventriculoarterial connections
Step 5	Assessment of the associated abnormalities at each segmental level

## 2. Contemporary sequential segmental approach, illustrated case examples

### 2.1. Determination of the visceral and atrial situs

**Figure 1:**
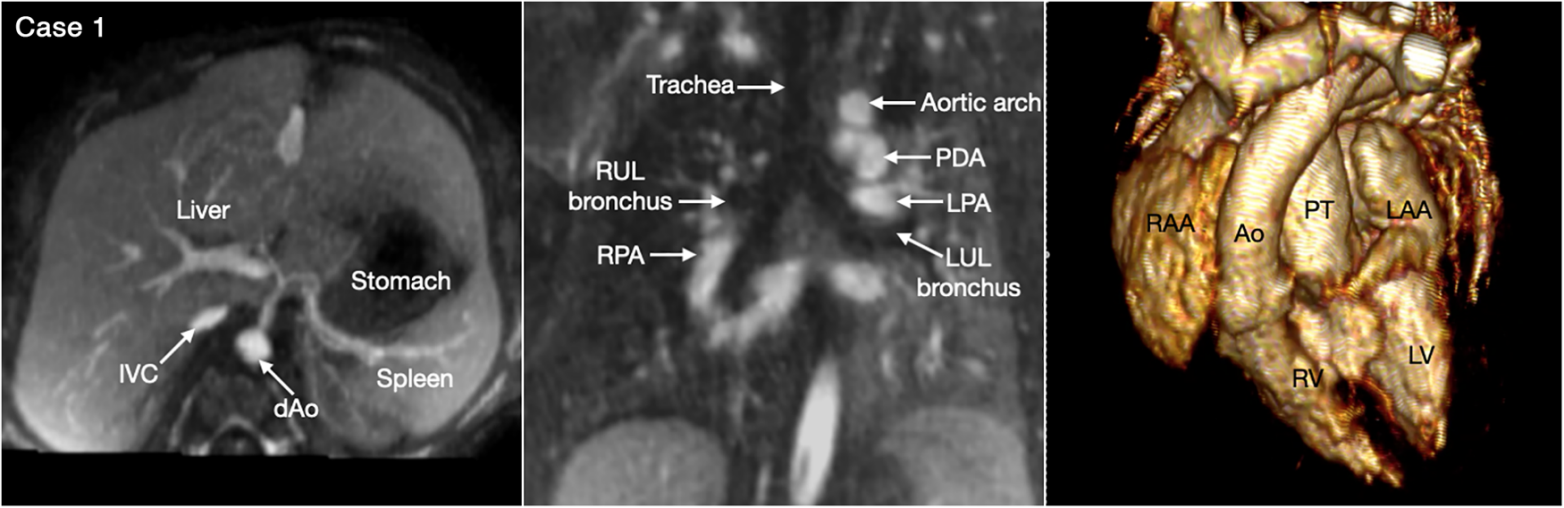
(Case 1). Normal arrangement of the abdominal organs, bronchopulmonary branching and atria in a patient with double outlet right ventricle and a subpulmonary ventricular septal defect. The abdominal situs is determined by observing the position of the liver, stomach and spleen. It is also important to observe the positions of the inferior vena cava (IVC) and the abdominal descending aorta (dAo). The bronchopulmonary situs is determined by observing the relationship between the upper lobar bronchus and the pulmonary arterial branch. The atrial situs is determined by observing the shape of the atrial appendages. LAA, left atrial appendage; LPA, left pulmonary artery; LUL, left upper lobe; LV, left ventricle; PDA, patent ductus arteriosus; PT, pulmonary arterial trunk; RAA, right atrial appendage; RUL, right upper lobe; RV, right ventricle.

Situs refers to the pattern of arrangement of the solid organs relative to the midline or sagittal plane of the body (17, 18). To determine the situs, organ arrangement is observed at three levels; abdomen, bronchopulmonary branching and atria. While the situs at all three levels is harmonious in the majority of cases, approximately 20% of cases having abnormal situs show disharmonious situs patterns at three levels (18). Therefore, the situs should be determined separately for the abdominal organs, the bronchopulmonary arrangement, and the atria (Figure 1).

**Figure 2:**
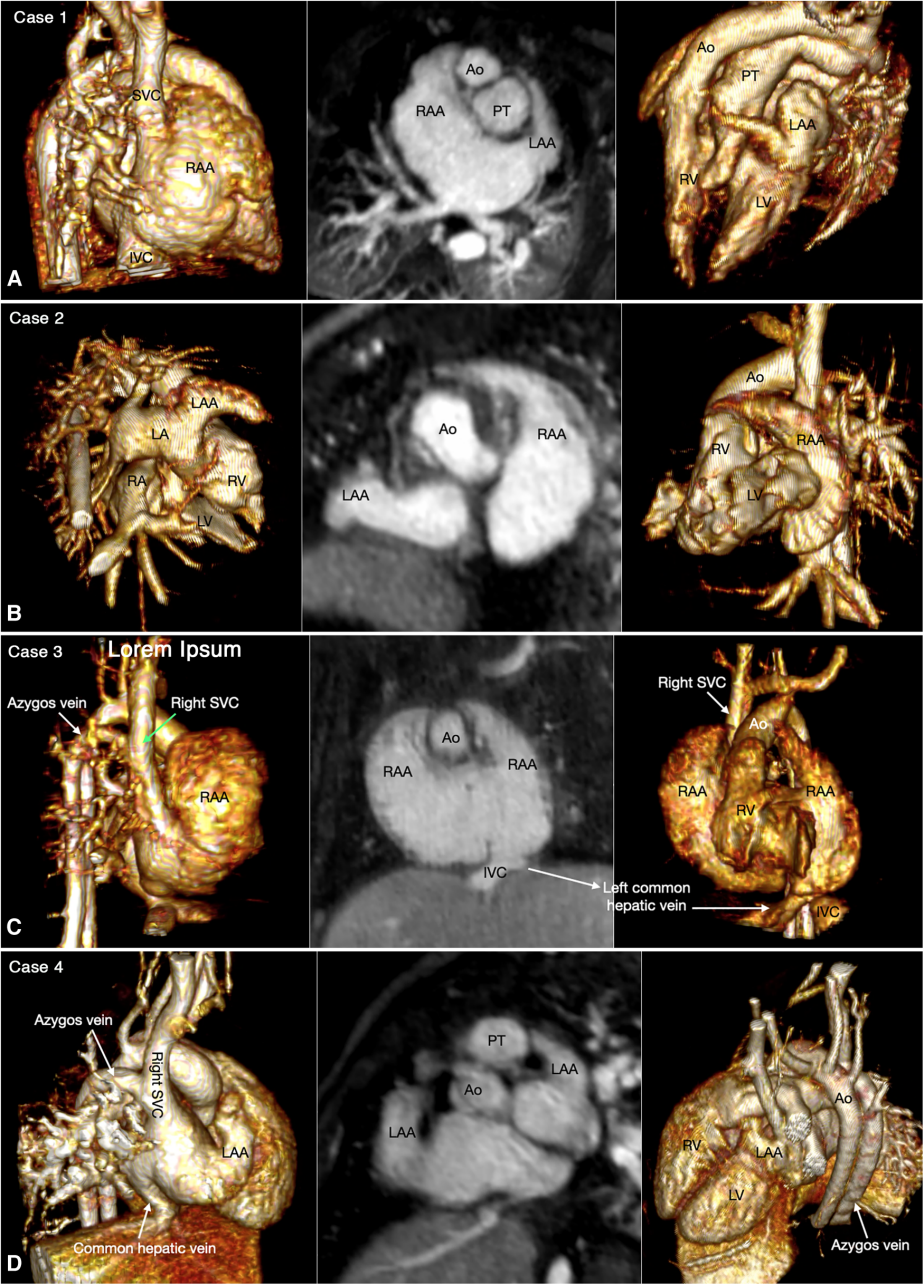
Four classic patterns of atrial situs: atrial situs solitus (**A**, case 1), atrial situs inversus (**B**, case 2), atrial right isomerism (**C**, case 3) and atrial left isomerism (**D**, case 4). The left and right panels are volume rendered images taken in oblique views for demonstration of the right-sided and left-sided atrial appendages, respectively. The middle panels are maximum intensity projection images obtained in short axis of the heart for visualization of both appendages. The morphology of the atria is determined by the shape of the appendage and the extent of the pectinate muscles that demarcate the boundary of the appendage. IVC, inferior vena cava; LA, left atrium; LAA, left atrial appendage; LV, left ventricle; RA, right atrium; RAA, right atrial appendage; RV, right ventricle; SVC, superior vena cava.

Abdominal and bronchopulmonary situs is easy to determine by observing the liver, stomach, spleen, airways and pulmonary arterial branches at cross-sectional imaging (Figure 1, Case 1). The atrial situs is determined by observing the shape of the atrial appendages. With some limitation, the shapes of the atrial appendages are better appreciated using volume rendered (VR) than sectional images, particularly when the VR images are displayed in cine mode (Figure 2 Cases 1–4). When the shapes of the appendages are not clearly identifiable, the distribution of the pectinate muscles along the parietal wall of the atria is helpful (19, 20). As the morphologically right atrial appendage has a wide junction with the body of the atrium, the pectinate muscles of the right atrial appendage are distributed all around the parietal aspect of the atrial wall close to the outlet or vestibule of the atrium. Because of the narrow junction of the morphologically left atrial appendage with the body of the atrium, the pectinate muscles of the left atrial appendage are identifiable in a limited part of the parietal aspect of the atrial wall. The distribution of the pectinate muscles is more easily defined in cine display of the thin MIP images than in static images (middle panels in Figure 2).

### 2.2. Juxtaposition of the atrial appendages

**Figure 3:**
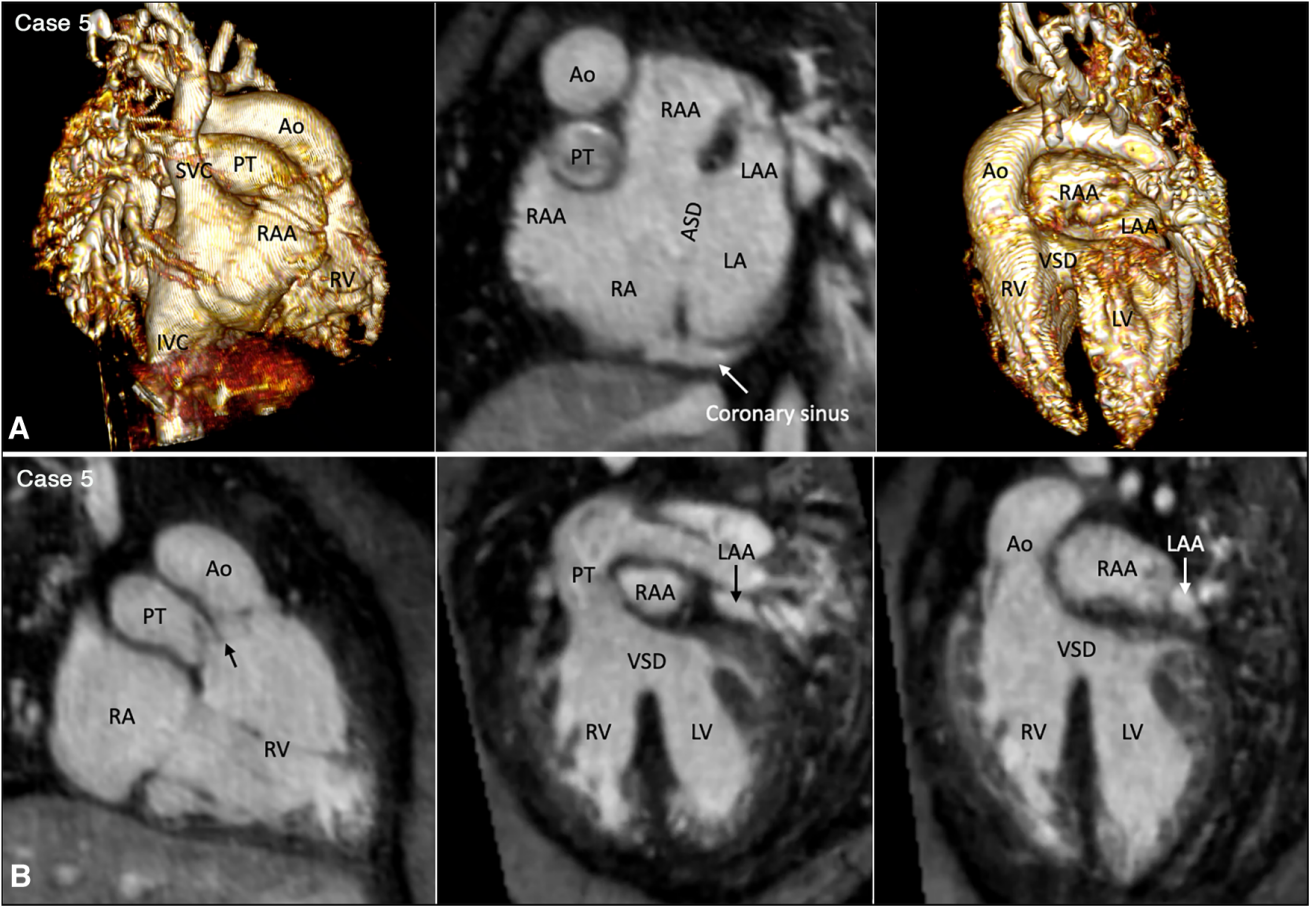
(Case 5). Partial left juxtaposition of the atrial appendages in a patient with situs solitus, concordant atrioventricular connection and double outlet right ventricle. (**A**) Atrial appendages shown in volume rendered (VR) images taken in oblique views (left and right panels) and maximum intensity projection (MIP) images in short axis plane (middle panel). A part of the right atrial appendage (RAA) is displaced to the left side to lie above and medial to the left atrial appendage (LAA). There is a large atrial septal defect (ASD) in the obliquely oriented atrial septum. (**B**) MIP images obtained in right anterior oblique view of the right ventricle (left panel) and long axial oblique views of the ventricles (middle and right panels) showing double outlet right ventricle (RV) with a large ventricular septal defect (VSD) at a distance from the valves of the pulmonary arterial trunk (PT) and aorta (Ao). The aortic valve is supported by a long muscular infundibulum and the pulmonary valve is supported by a short muscular infundibulum. The aortic and pulmonary valves are in fibrous continuity above the vestigial outlet septum (arrow in left panel). IVC, inferior vena cava; LA, left atrium; LV, left ventricle; RA, right atrium; SVC, superior vena cava.

**Figure 4:**
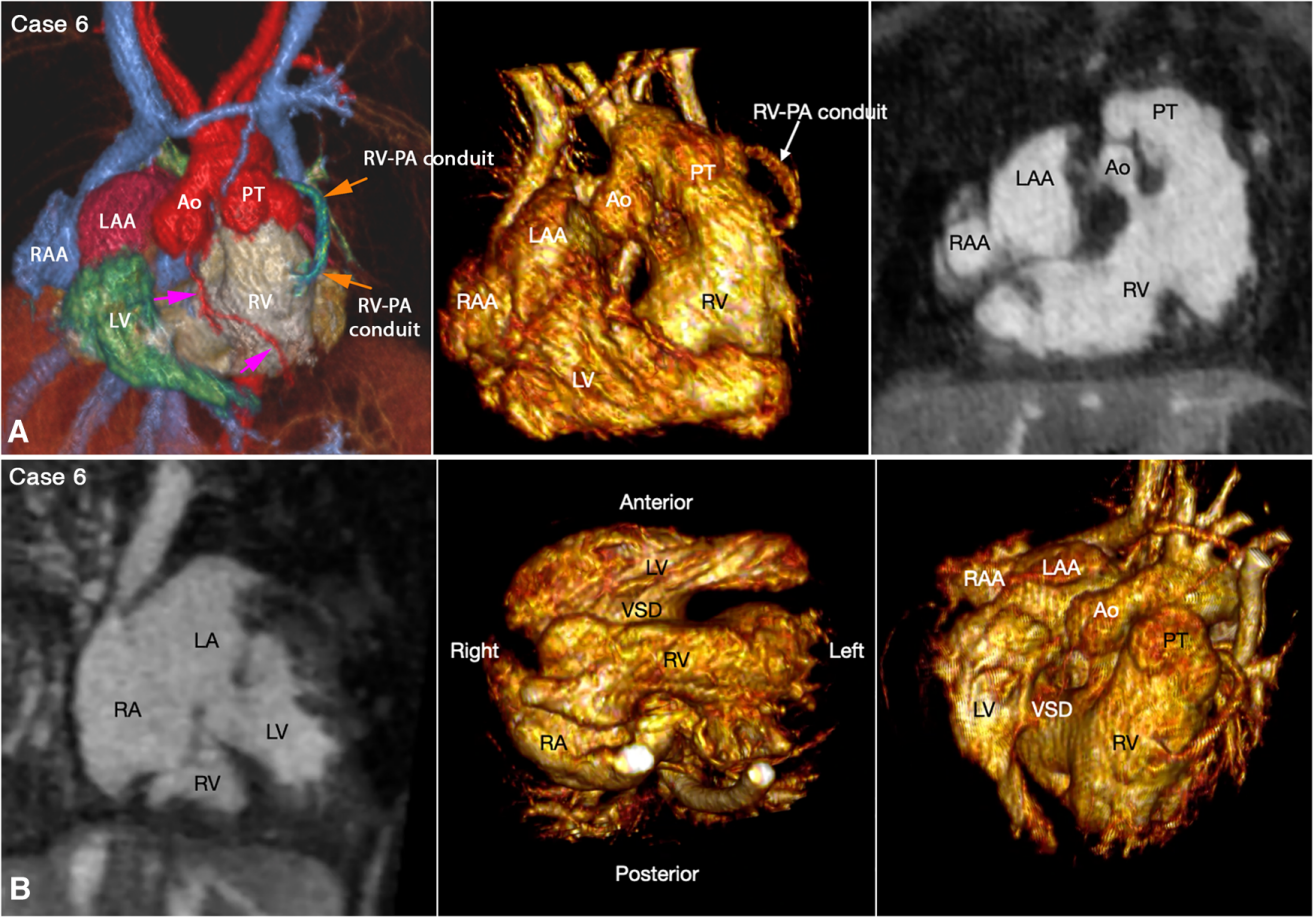
(Case 6). Right juxtaposition of the atrial appendages in a patient with situs solitus, concordant atrioventricular connection to the L-looped ventricles and double outlet right ventricle. (**A**) Atrial appendages shown in volume rendered (VR) images (left and middle panels) and maximum intensity projection (MIP) images in short axis plane (right panel) taken in right anterior oblique views. The left atrial appendage (LAA) is displaced to the right side of the arterial trunks and is placed above and medial to the right atrial appendage (RAA). (**B**) MIP image obtained in a parasagittal oblique view across the mitral and tricuspid valves (left panel), VR image seen from below (middle panel) and VR image obtained from the ventricular apex with the ventricular septum oriented vertically (right panel). The left atrium (LA) is connected to the anteriorly located left ventricle (LV), and the right atrium (RA) is connected to the right ventricle (RV) that wraps around the left ventricle from behind. While the bloodstreams are crossed within the atrial segment, the mitral and tricuspid valve axes are parallel as shown in left panel. Right panel shows the ventricular relationship that is seen in hearts with L-looped ventricles. There is a large perimembranous ventricular septal defect (VSD). Pink arrows indicate the anterior descending coronary artery demarcating the junction between the right and left ventricles (This case was previously reported. Perens G, Takegawa Y, Finn PJ. Congenital Heart Disease 2022;17(4):387-392. doi: 10.32604/chd.2022.021233).

Juxtaposition of the atrial appendages on either side of the arterial trunks is uncommon and usually occurs as part and parcel of complex malformation (21, 22). Juxtaposition can involve the entire or part of the tip of the appendage for which the terms “complete” and “partial” juxtapositions have been introduced. The displaced part of the appendage is always on top of the normally positioned appendage. Usually, the appendage of the morphologically right atrium is displaced to the other side to lie on top of the appendage of the morphologically left atrium. Less commonly, the appendage of the morphologically left atrium is displaced to the other side to lie on top of the appendage of the morphologically right atrium. Juxtaposition also occurs in association with heterotaxy (22). Both complete and partial juxtaposition can well be appreciated at cine VR display (Figures 3,4 Cases 5 and 6). Juxtaposition of the right atrial appendage above the left atrial appendage including left juxtaposition in situs solitus and right juxtaposition in situs inversus occurs most commonly with double outlet right ventricle, transposition of the great arteries and tricuspid atresia (21–23). Juxtaposition of the left atrial appendage above the right atrial appendage including right juxtaposition in situs solitus and left juxtaposition in situs inversus is associated with exceedingly rare complex malformation (24, 25). Right juxtaposition was also reported to occur in otherwise normal heart or minor cardiac anomalies (26). When there is juxtaposition of the atrial appendages, the atrial septum shows distorted orientation, and the atrioventricular valve of the affected atrium is mildly or grossly displaced (27).

### 2.3. Systemic and pulmonary venous connections

Clear definition of the venous connections to the atria is particularly important in patients with heterotaxy as rerouting of the systemic and pulmonary venous circulation is required not only for biventricular but also for univentricular repair. From the imaging perspective, 3D imaging with a wide field of view is helpful for the assessment of the venous abnormalities. MR angiography is certainly advantageous over CT angiography as proper contrast enhancement of both systemic and pulmonary venous structures is a difficult task at CT angiography.

**Figure 5:**
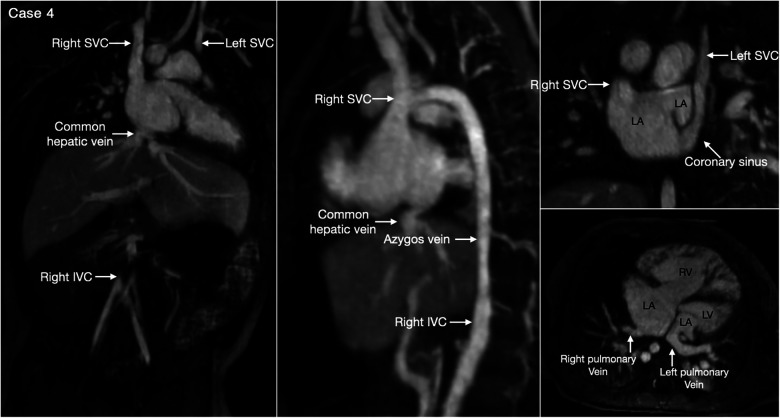
(Case 4). Bilateral superior venae cavae and interrupted inferior vena cava in a patient with heterotaxy and left isomerism. There is no bridging vein between the two superior venae cavae (SVC). The left SVC connects to the coronary sinus that drains to the right-sided left atrium (LA). The right-sided inferior vena cava (IVC) is interrupted and continues to the right-sided SVC. The right and left pulmonary veins connect to the posterior wall of the ipsilateral left atrium. There is a large defect involving the most superior part of the atrial septum. There is myocardial noncompaction of the left ventricle (LV). RA, right atrium; RV, right ventricle.

**Figure 6:**
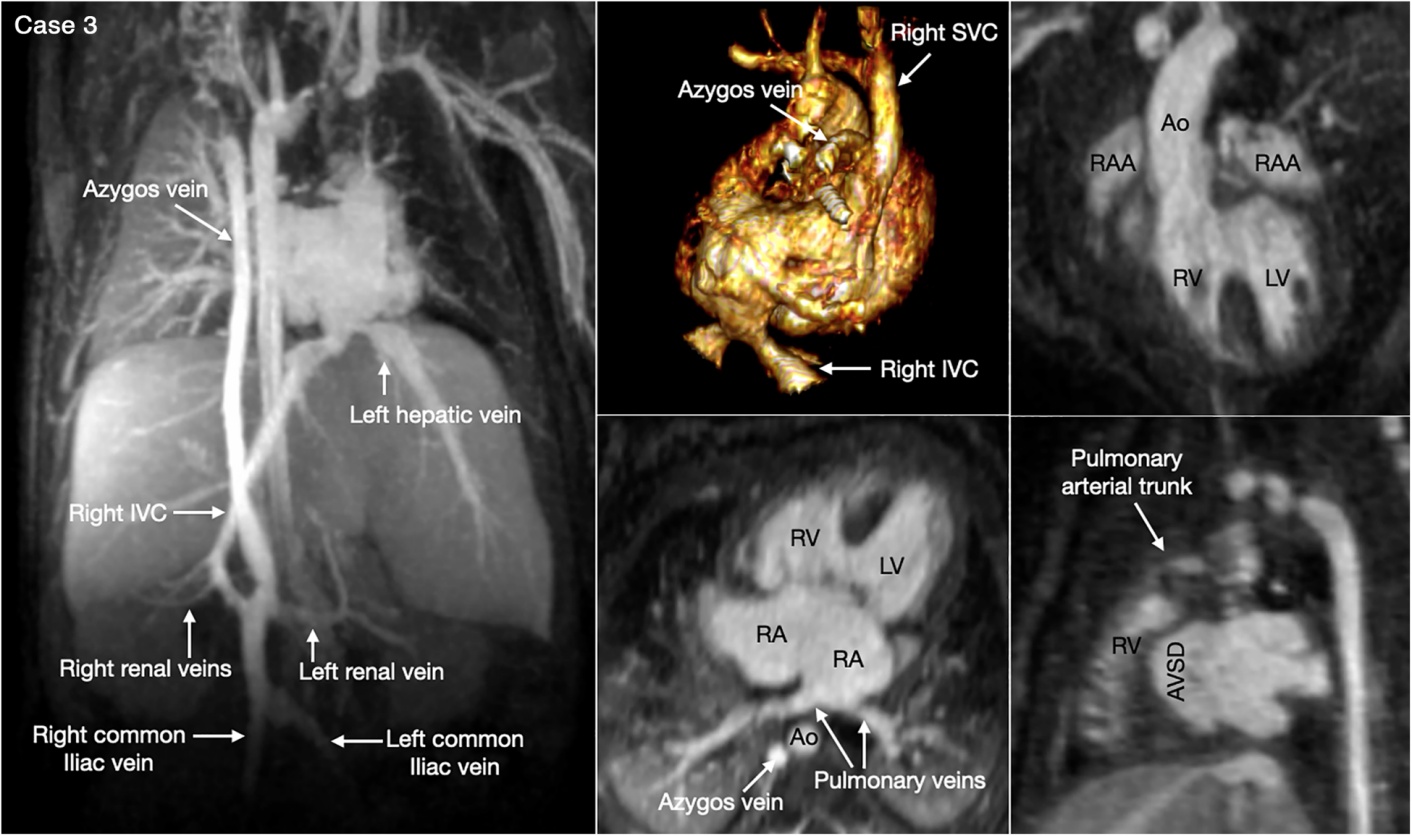
(Case 3). Complex systemic venous anatomy in a patient with heterotaxy and right isomerism. Left and middle upper panels show the systemic venous anatomy. The right inferior venae cava (IVC) gives rise to a large branch that drains to the dilated right-sided azygos vein that then connects to the right superior vena cava (SVC). The right inferior vena cave ascends obliquely through the liver parenchyma taking hepatic veins from the right lobe of the liver and then connects to the left-sided atrium. The left hepatic vein also connects to the left-sided atrium near the inferior vena caval connection. Middle and right lower panels show a large atrioventricular septal defect (AVSD) and the pulmonary veins connected to the left-sided right atrium. There is malalignment between the atrial and ventricular septa. Right panels show both the aorta (Ao) and small pulmonary arterial trunk arising from the morphologically right ventricle (RV). There are right isomeric atrial appendages (RAA). LV, left ventricle; RA, right atrium.

**Figure 7:**
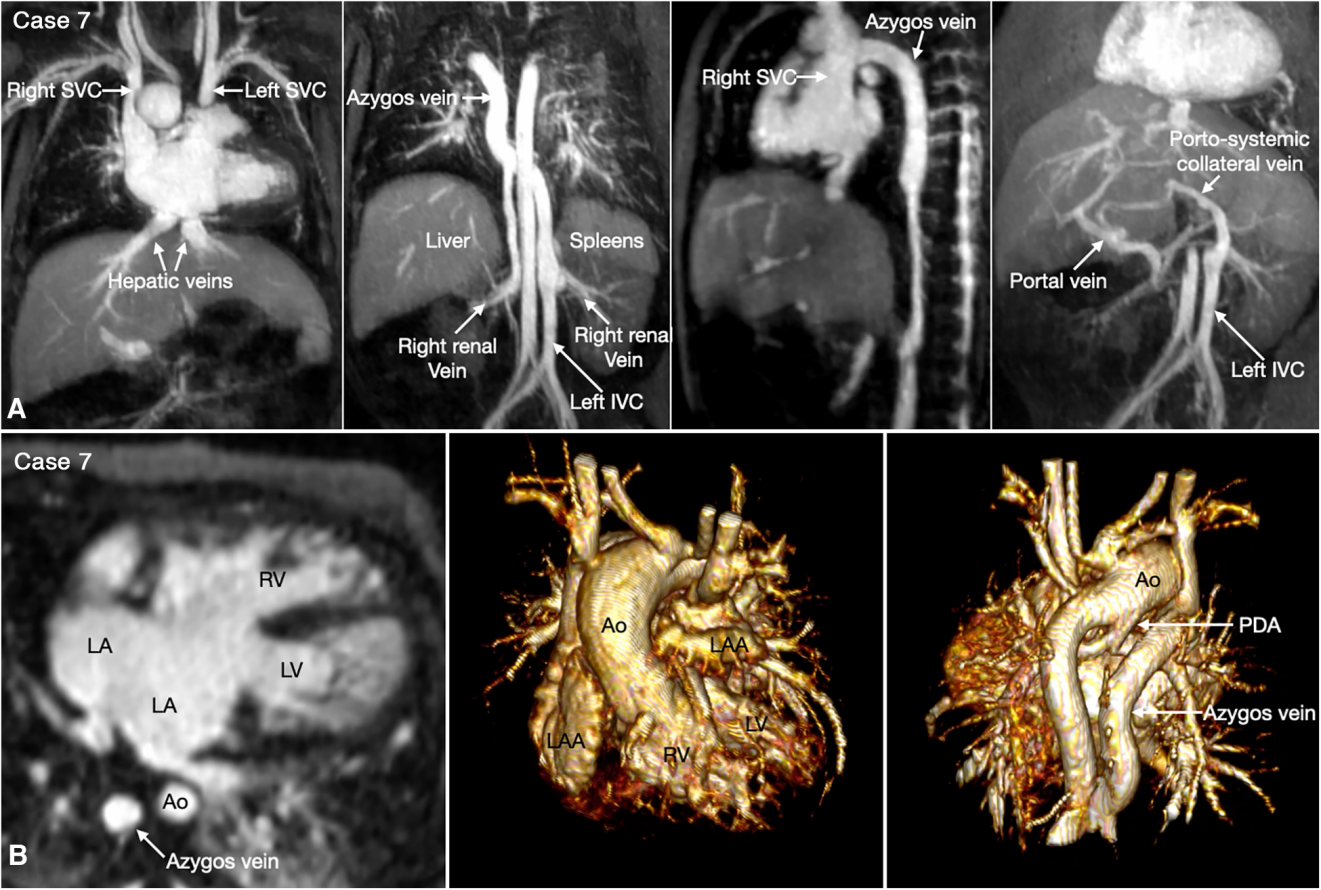
(Case 7). Complex systemic venous anatomy and extrahepatic portosystemic shunt in a patient with heterotaxy and left isomerism. (**A**) Maximum intensity projection (MIP) images show that the left inferior vena cava (IVC) is interrupted and continues to the left-sided (hemi)azygos vein. It then obliquely ascends to the right-sided azygos vein that connects to the right superior vena cava (SVC). The right renal vein also connects to the right-sided azygos vein through a vertical channel. Right panel shows a tortuous collateral channel connecting the unobstructed portal vein and the inferior vena cava. (**B**) MIP image in 4-chamber plane and volume rendered images in frontal and posterior superior views. Middle panel shows bilateral morphologically left atrial appendages (LAA). Left panel shows a large atrioventricular septal defect. The pulmonary veins are connected to the posterior wall of the common atrium. Middle panel shows both aorta (Ao) and severely stenotic pulmonary arterial trunk arising from the right ventricle (RV). Right panel shows a patent ductus arteriosus (PDA) arising from the undersurface of the aortic arch and connecting to the confluent pulmonary artery. LA, left atrium; LV, left ventricle.

The most common is bilateral superior venae cavae with or without a bridging vein (Figure 5, Case 4). The venous anatomy is unpredictable when there is heterotaxy (Figures 5–7, Cases 3, 4, 7) (18, 28). Interruption of the inferior vena cava with azygos or hemiazygos continuation is seen in >80% of heterotaxy cases with left isomerism, while extracardiac total anomalous pulmonary venous connection is sees in >50% of heterotaxy cases with right isomerism. The location of the superior vena cava and its connection site are important in planning cardiopulmonary bypass and establishing a bidirectional cavopulmonary anastomosis when needed. Precise depiction of the sites of the systemic and pulmonary venous connections is also very important for surgical separation of the systemic and venous returns within the atrium.

**Figure 8:**
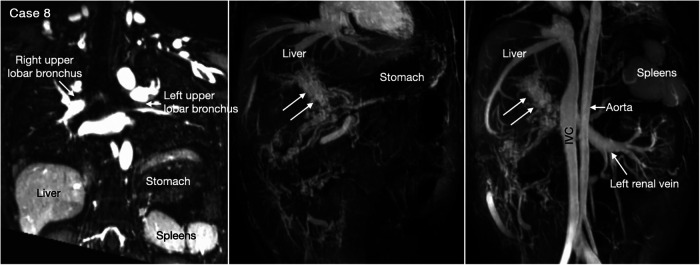
(Case 8). Cavernous transformation of the portal vein in a patient with left-sided polysplenia and otherwise normal body and atrial situs. Left panel shows bronchopulmonary situs solitus, and left-sided stomach and polysplenia. Middle and right panels show cavernous vascular plexus (two white arrows) along the expected course of the portal vein. Cavernous channels are also seen along the intestine. Right panel shows dilated renal vein functioning as portosystemic venous channel. Although cavernous transformation typically develops as a result of portal vein thrombosis, the association of polysplenia in this patient suggests that cavernous transformation is associated with congenital agenesis or hypoplasia of the portal vein. IVC, inferior vena cava.

Heterotaxy with left isomerism is often associated with extrahepatic portosystemic shunt (29, 30). As the portosystemic shunt results in chronic liver disease with portal hypertension and hepatic encephalopathy, its early recognition is important. 3D or 4D MR angiography is very helpful in visualization of the portal venous anatomy and collateral pathways owing to its wide field of view and homogeneous opacification of the vessels (Figures 7,8 Cases 7 and 8).

### 2.4. Determination of the ventricular relationship and atrioventricular connections

**Figure 9:**
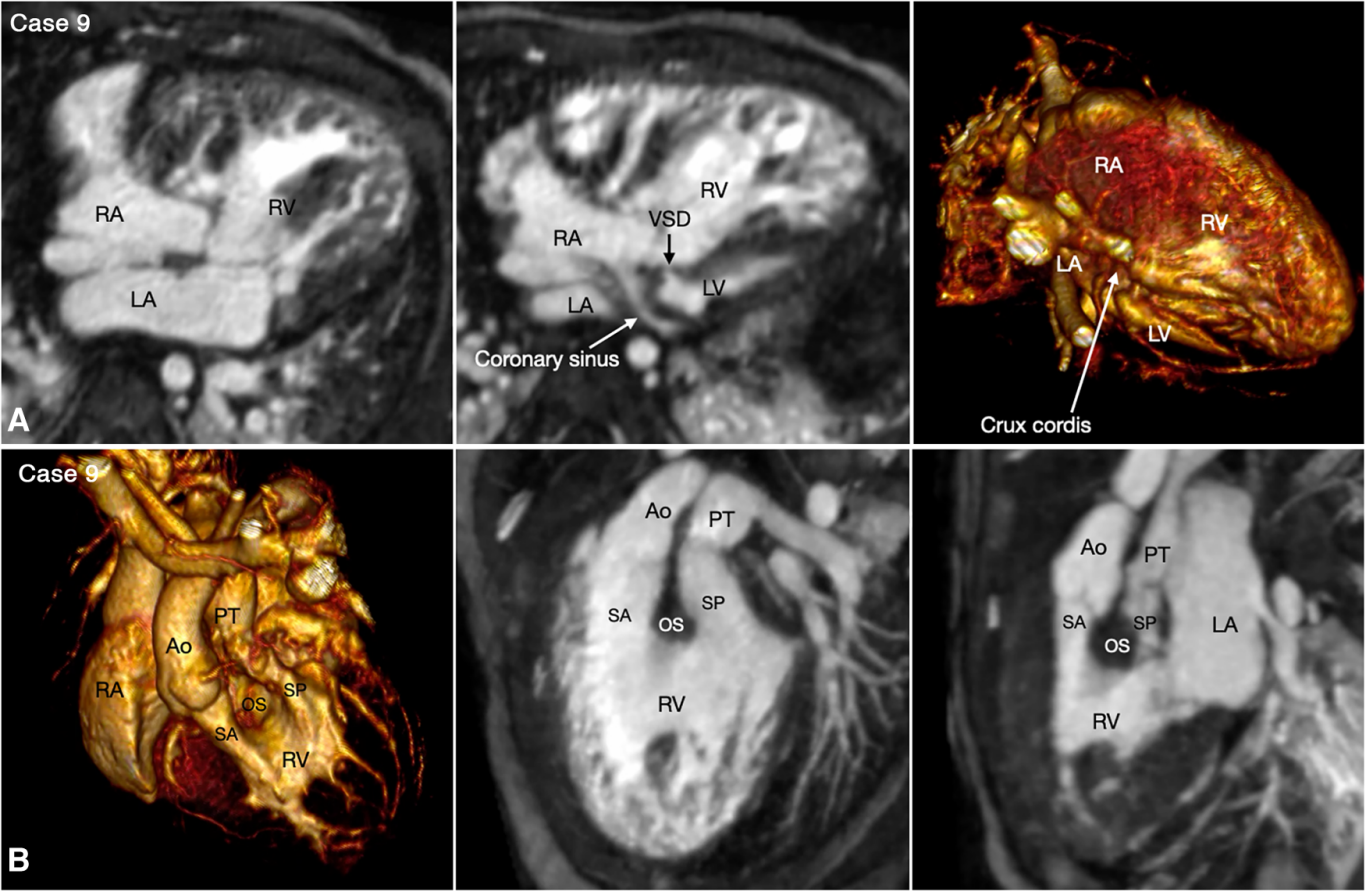
(Case 9). Double inlet and double outlet right ventricle in a patient with situs solitus and levocardia. (**A**) Maximum intensity projection images (MIP) in 4-chamber planes (left and middle panels) and volume rendered (VR) image (right panel) seen from below simulating the view in the middle panel. Both right (RA) and left (LA) atria connect to the main chamber of right ventricular morphology (RV). The severely hypoplastic left ventricle (LV) is positioned along the diaphragmatic surface at the left corner of the crux cordis. (**B**) VR images in left anterosuperior view (left panel) and MIP images in steep left anterior oblique planes (middle and right panel). The VR image and MIP image in right panel were obtained in end systole and the MIP image in the middle panel was obtained in end diastole. Both aorta (Ao) and pulmonary arterial trunk (PT) arise from the right ventricle. The right ventricular outflow tract is divided into the subaortic (SA) and subpulmonary ((SP) outflow tracts by the hypertrophied outlet septum (OS). Both outflow tracts show severe dynamic narrowing in systole. The aorta is positioned on the right anterior aspect of the pulmonary arterial trunk.

**Figure 10:**
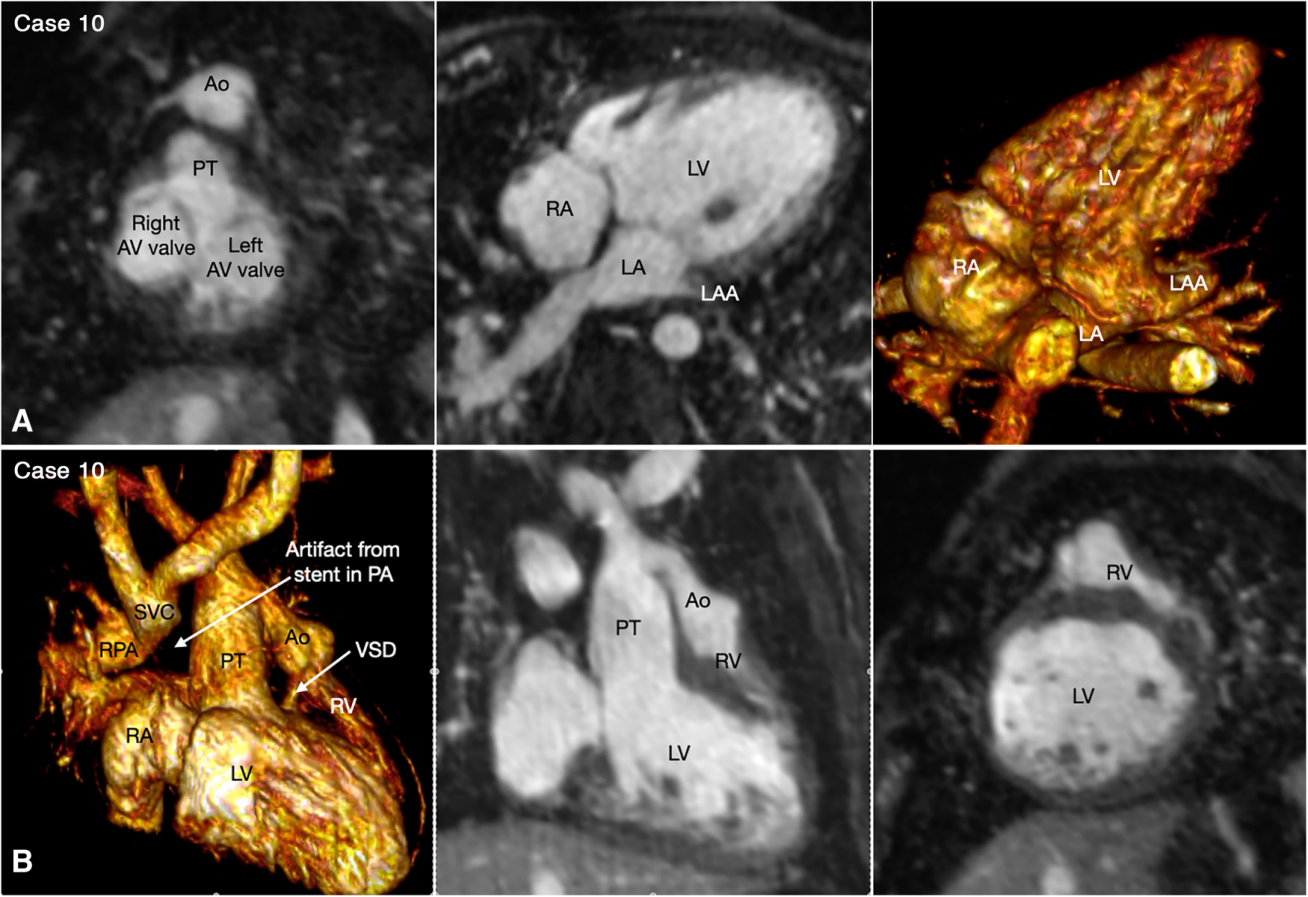
(Case 10). Double inlet left ventricle with discordant ventriculoarterial connection, status post Damus-Kaye-Stensel (DKS) procedure and aortic arch reconstruction followed by bidirectional cavopulmonary anastomosis. A short-axis maximum intensity projection (MIP) image (**A**-left panel) shows equally sized right and left atrioventricular (AV) valves. MIP image in an oblique axial image and volume rendered (VR) image taken in a similar view (A, middle and right panels) show that both atria are connected to the main chamber of left ventricular morphology (LV). VR and MIP images taken in a right anterior oblique projection and a short-axis MIP image in (**B**) show that the incompletely formed, hypoplastic right ventricle (RV) is located superiorly and to the left in relation to the left ventricle. The ventricular septal defect (VSD) is very small. The native aorta (Ao) arises from the right ventricle and the native pulmonary arterial trunk (PT) arise from the left ventricle. The aortic valve and root are located at the left and anterior aspect of the pulmonary valve and arterial trunk.

With rare exceptions, the ventricular mass consists of two ventricles, one morphologically right and the other morphologically left. When the two ventricles are well developed, the ventricular morphology can easily be identified by using the criteria including the overall shape of the ventricle, the trabeculation pattern, the presence or absence of the moderator band and the level of attachment of the atrioventricular valve to the septum (Figures 3–7, Cases 3–7). When one ventricle is incompletely formed and hypoplastic, these criteria may not be applicable or difficult to apply. On imaging, the relative position of the ventricles provides the accurate and most reliable clue to their morphology (27, 31, 32). The hypoplastic left ventricle is almost always located along the inferior diaphragmatic surface of the heart at either side of the crux cordis (Figure 9, Case 9). The incompletely formed right ventricle, however, is almost always located anteriorly and superiorly away from the crux cordis (Figure 10, Case 10). When there is only one ventricular chamber identifiable, the ventricular mass is described as the indeterminate ventricle. However, the solitary ventricle usually shows the classic characteristics of the morphologically right ventricle, suggesting that the severely hypoplastic left ventricle is not identifiable rather than not present. Rarely, the solitary ventricle may appear to be a common ventricular chamber because of almost complete absence of the ventricular septum. Cine display of the VR images is an easy mean for the morphological identification of the ventricles, particularly when the ventricular mass consists of a main ventricular chamber and a rudimentary ventricle (Figures 9, 10, Cases 9 and 10).

The relationship between the two ventricles is classified into D-loop (the right ventricle on the right side of the left ventricle) and L-loop (the right ventricle on the left side of the left ventricle) ventricular relationships. In the majority of cases, the right-left relationship is described by referencing the right-left coordinates of the body (Figures 3–7, Cases 3–7). Uncommonly, the right-left relationship of the ventricles is grossly distorted with the ventricles superoinferiorly related or the atrioventricular connections twisted (33). Despite the distorted spatial orientation in these exceptional cases, however, the internal anatomic orientation of the ventricles is not changed, and the relationship between the ventricles relative to the ventricular septum is maintained. Therefore, the D- or L-loop ventricular relationship can be defined by observing the positions of the right and left ventricles in relation to the ventricular septum in a neutral position (34). In imaging, the ventricular mass is rotated to a position where the ventricular septum is vertically oriented, the ventricular apex faces the observer and the arterial trunks are directed upward, namely the “ventricular apical view” (Figure 4B, Case 6) (33).

Once the atrial and ventricular relationship are determined, the atrioventricular connection is assessed. The most helpful for assessment of the atrioventricular connections is cine display of the thin MIP and VR images. In the vast majority of cases, the two atrioventricular connection axes are parallel and the type of atrioventricular connection is predictive of the relationship of the underlying ventricles. Therefore, the atrioventricular connections are usually readily appreciated in 4-chamber plane regardless of whether there are biventricular or univentricular atrioventricular connections (Figures 3–7, 9, 10, Cases 3–7 and 10). When there are unexpected spatial relationships of the cardiac chambers and arterial trunks for the given segmental connections as discussed above, the atrioventricular connection is assessed using multiplanar MIP images along each atrioventricular valve and VR images (Figure 4B, Case 6).

### 2.5. Determination of the great arterial relationship and ventriculoarterial connection

**Figure 11:**
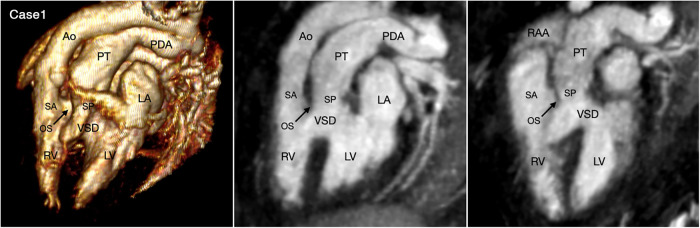
(Case 1). Double outlet right ventricle with a subpulmonary ventricular septal defect, so-called Taussig-Bing malformation. Volume rendered (VR) image and maximum intensity projection (MIP) images in long axial oblique projection demonstrate the aorta (Ao) arising from the right ventricle (RV) through a long subaortic (SA) infundibulum and the pulmonary arterial trunk (PT) arising from both ventricles through a shorter subpulmonary (SP) infundibulum. The outlet septum (OS) is displaced forward into the right ventricle. While the severity of pulmonary valve overriding is well appreciated at 3D VR image, it is different in 2D MIP images depending on the imaging plane. LA, left atrium; LV, left ventricle; RAA, right atrial appendage.

The great arterial relationship is determined by describing the position of the aortic root and valve in relation to the pulmonary arterial trunk and valve. The ventriculoarterial connection is unequivocally definable when the ventricular septum is intact. When there is a ventricular septal defect underneath one or both arterial valves, the type of ventriculoarterial connection can be controversial as the ventricular septum is not uniformly oriented in relation to the arterial valves (35). Although the “50% rule” is used to define the ventriculoarterial connection when there is an overriding arterial valve, it is often difficult to apply in cross-sectional imaging when the arterial valve may appear to arise predominantly from one ventricle in one imaging plane, while the same valve may appear to arise predominantly from the other ventricle in other imaging plane. We find the ventriculoarterial connection is more consistently definable in VR images than in cross-sectional images (Figures 3,10, 11, Cases 5, 10, and 1).

### 2.6. Ventricular outflow tract obstruction

**Figure 12:**
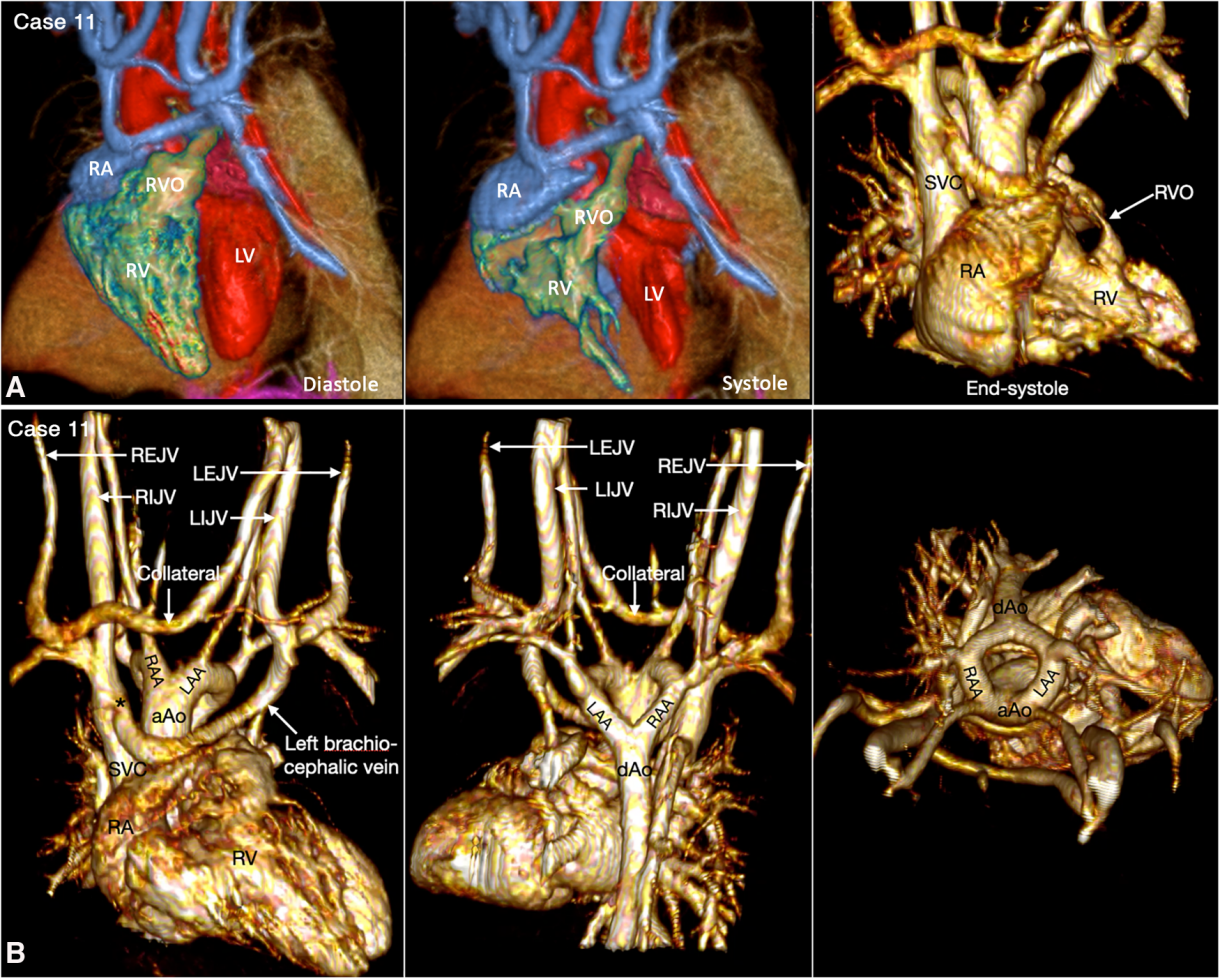
(Case 11). Dynamic right ventricular outflow tract narrowing in a patient with tetralogy of Fallot. (**A**) Volume rendered (VR) images taken in diastole (left panel) and end-systole (middle and right panel) show severe dynamic subpulmonary right ventricular outflow tract (RVO) narrowing is best appreciated in lateral image taken in end-systole. (**B**) VR images showing double aortic arch. The left brachiocephalic vein shows narrowing (asterisk) at its connection to the superior vena cava (SVC). A collateral channel is seen between the left internal jugular vein (LIJV) and the right external jugular vein (REJV). aAo, ascending aorta; dAo, descending aorta; LAA, left aortic arch; LEJV, left external jugular vein; LV, left ventricle; RA, right atrium; RAA, right aortic arch; RIJV, right internal jugular vein; RV, right ventricle; VSD, ventricular septal defect.

The ventricular outflow tract obstruction can be a fixed lesion but, more commonly, a dynamic lesion. The dynamic ventricular outflow tract obstruction cannot be accurately assessed using static imaging technique. It is also difficult to clearly demonstrate the exact severity of dynamic narrowing at 2D cine imaging as the region of the interest moves in and out of the imaging plane during the cardiac cycle. 4D VR imaging is particularly helpful as the dynamic nature of the obstruction can be observed throughout the cardiac cycle (Figures 9, 12, Cases 9 and 11).

### 2.7. Coronary arterial abnormalities

**Figure 13:**
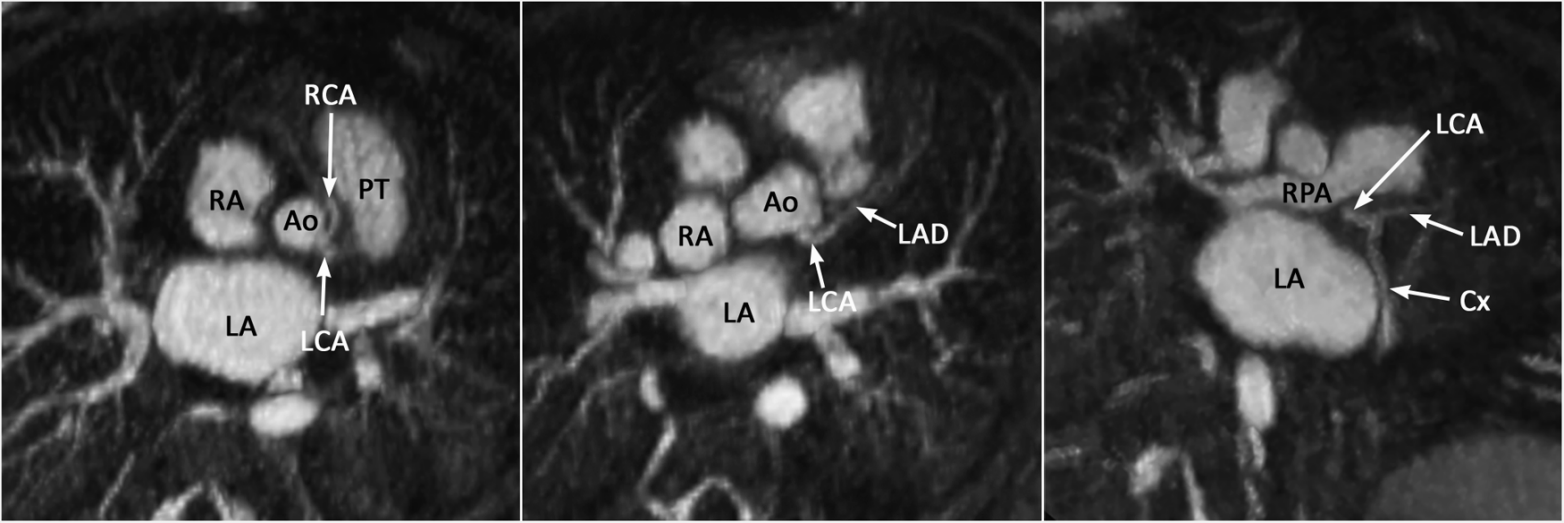
(Case 12). Common origin of the left (LCA) and right (RCA) coronary arteries from the sinotubular junction above the left coronary sinus. The RCA takes an interarterial course. An acute angle of its origin is highly suggestive of an intramural course. Ao, aorta; Cx, circumflex coronary artery; LA, left atrium; LAD, left anterior descending coronary artery; PT, pulmonary arterial trunk; RA, right atrium.

Proper identification of the origins and courses of a coronary artery or arteries are important for preoperative assessment for arterial switch operation, Ross operation and right ventricular outflow tract reconstruction. Detection of an abnormal interarterial and/or intramural course and high take-off of a coronary artery is important as it may result in sudden cardiac death. With some limitation, the coronary arterial origins and courses can be accurately assessed with cine display of MIP images (Figure 13). It should be noted that whereas 4D MUSIC can define coronary anatomy in most case, it may not do so in all cases. Where doubt persists, coronary CT angiography should be considered.

## 3. Conclusion

MR imaging using the 4D MUSIC technique following intravenous administration of ferumoxytol provides a single data set for the assessment of dynamic cardiovascular anatomy and ventricular function at the same time. Owing to a strong T1-shortening effect and a long half-life of ferumoxytol, 4D MUSIC imaging enables data acquisition with electrocardiographic and respiratory gating for a high-resolution 4D acquisition that takes several (8–12) minutes. Homogeneous opacification of all cardiovascular structures within the imaging volume allows full sequential segmental approach to the congenital heart diseases without any blind spots. The complex systemic and pulmonary venous anatomy is particularly well captured in 4D MUSIC imaging when compared to other conventional techniques. Cinematographic display of multiplanar sectional and 3D volume images is helpful in the morphological identification of the cardiac chambers, the assessment of the dynamic nature of the ventricular outflow tracts, and the assessment of the coronary arterial origins and courses. Because acquisition takes several minutes, the image quality relies heavily on successful mitigation of cardiac and respiratory motion artifact. To date, limited access to ferumoxytol for off-label diagnostic use, as well as its high cost, have posed obstacles to the more widespread utilization of the 4D MUSIC technique. However, the hope is that these limitations will subside as generic versions become available within and beyond the U.S.
